# Accessibility, Inclusion, and Rehabilitation Using Information Technologies

**DOI:** 10.1155/2020/9460536

**Published:** 2020-02-21

**Authors:** Antoni Jaume-i-Capó, Yolanda González-Cid, Anthony L. Brooks, Stuart Cunningham

**Affiliations:** ^1^Unitat de Gràfics i Visió per Computador i Intel·ligència Artificial, Departament de Ciències Matemàtiques i Informàtica, Universitat de les Illes Balears, Palma, Spain; ^2^Sistemes, Robòtica i Visió, Departament de Ciències Matemàtiques i Informàtica, Universitat de les Illes Balears, Palma, Spain; ^3^Department of Architecture, Design, & Media Technology, Aalborg University, Aalborg, Denmark; ^4^Department of Computing and Mathematics, Manchester Metropolitan University, Manchester, UK

Social exclusion occurs when individuals, or even entire communities of people, are blocked from rights, opportunities, and resources, preventing them from fully participating in the activities of the society in which they live.

After the success of AIRTech2018 (the second edition of the International Conference on Accessibility, Inclusion and Rehabilitation using Information Technologies), we, the editors, proposed to this special issue the *Journal of Healthcare Engineering*. The purpose of this special issue is to publish recent advances in developmental accessibility, inclusion, and rehabilitation using Information Technologies. The term Information Technologies in context to this special issue refers in general to the development and use of computer systems, software, and networks in relation to issues of accessibility, inclusion, and/or rehabilitation. The articles received reflects advancements and opportunities brought about in this field through technical innovations alongside adoption and inquiry by those practicing and/or researching in this important field associated with healthcare and societal wellbeing.

The quality level of the submissions for this special issue was very high. A total of 11 manuscripts were submitted to this issue in response to the call for papers. Based on a rigorous review process, 5 papers were accepted for publication in the special issue. Below, we briefly summarize the highlights of each paper.

## 1. The Special Issue

Cunningham et al. address the demographic time bomb of caring for people living with dementia. Their study introduces a care platform named *Memory Tracks*, which utilizes reminiscence music and song-task association in an attempt to improve the wellbeing of people living with dementia and those caring for them. Initial indicators regarding the efficacy of the platform are presented following a mixed-method study with a small cohort living with dementia in a care home, between levels 5 and 6 on the Global Deterioration Scale for Assessment of Primary Degenerative Dementia.

Waliño-Paniagua et al. propose to analyze a virtual reality video capture training program plus occupational therapy on manual dexterity in patients with multiple sclerosis. Clinical improvements were found regarding the precision of movements, the execution times, and the efficiency of certain functional tasks in the Purdue Pegboard Test and Jebsen–Taylor Hand Function Test.

The next article is titled “Prediction of the Spinal Musculoskeletal Loadings during Level Walking and Stair Climbing after Two Types of Simulated Interventions in Patients with Lumbar Disc Herniation.” Kuai et al.'s contribution reports on a study on lower back pain where spinal musculoskeletal loadings, mapped from test subjects' mocap data, inform preoperative treatment and rehabilitation planning. From this, an outcome goal was contribution toward improving capacity of spinal load sharing during activities of daily living (ADLs) after surgical intervention.

Focal vibration has shown benefits in the rehabilitation of people who have neurological conditions. Li et al. present a study that investigates the effects that focal vibration in the upper limb muscles have upon the human sensorimotor cortex. Their approach to explore this is to perform a three-phase study (before, during, and after focal vibration) and to measure the electroencephalography (EEG) response in twenty healthy male subjects. Their work presents a series of findings that contribute to what is currently known about the impact that the use of focal vibration in this setting may have upon its users.

Xu et al. propose the concept of using musculoskeletal modeling to estimate muscular states during elbow flexor resistance training for bedridden patients, and it is mainly on the discussion of computational methods. The results demonstrate that the measuring system can correctly estimate the elbow joint angle when the forearm flexes or extends in the sagittal plane.

